# Compliance with a high-protein and energy-dense oral nutritional supplement in patients with disease-related malnutrition: a randomized open-label crossover trial

**DOI:** 10.3389/fnut.2023.1182445

**Published:** 2023-05-02

**Authors:** Miguel Leon-Sanz, Francisca Linares, Montserrat Gonzalo, María José Tapia, María Maiz-Jimenez, Marta Ruiz Aguado, Luis Lizán, Gabriel Olveira

**Affiliations:** ^1^Department of Endocrinology and Nutrition, Hospital Universitario Doce de Octubre, Madrid, Spain; ^2^Instituto de Investigación 1+12, Madrid, Spain; ^3^Facultad de Medicina, Universidad Complutense de Madrid, Madrid, Spain; ^4^Department of Endocrinology and Nutrition, Hospital Regional Universitario de Málaga, Málaga, Spain; ^5^IBIMA, Instituto de Investigación Biomédica de Málaga y Plataforma BIONAND, Málaga, Spain; ^6^CIBERDEM, Centro de Investigación Biomédica en Red de Diabetes y Enfermedades Metabólicas Asociadas, Instituto de Salud Carlos III, Málaga, Spain; ^7^Outcomes’10, Castellón de la Plana, Spain; ^8^Facultad de Medicina, Universidad Jaume I, Castellón de la Plana, Spain; ^9^Facultad de Medicina de Málaga, Universidad de Málaga, Málaga, Spain

**Keywords:** oral nutritional supplement, energy density, compliance, cost, nutritional status, gastrointestinal tolerance

## Abstract

**Introduction:**

Patient compliance with oral nutritional supplements (ONS) is not optimal for meeting energy and nutritional requirements in a high proportion of patients with disease-related malnutrition (DRM). Energy density or prescribed volume of ONS may impact compliance.

**Methods:**

A randomized, open-label crossover trial was conducted in outpatients with DRM to compare compliance with a high energy-dense ONS (edONS, 2.4 kcal/mL) and a reference ONS (heONS, 2.0 kcal/mL; NCT05609006). Patients were randomly assigned to two 8-week treatment sequences of four-weeks periods: edONS + heONS (sequence A) or heONS + edONS (sequence B). Patients daily reported the amount of product left over gastrointestinal tolerance and satisfaction with ONS. A non-inferiority analysis was performed to compare the compliance rate (percentage of consumed energy over the prescribed) for each period and sequence.

**Results:**

Fifty-three patients were assigned to sequence A and 50 to sequence B (55.7 ± 13.9 years, 37.0% female, 67.1% oncology patients). In sequence A, the compliance rates were 88.6% ± 14.3% vs. 84.1 ± 21.8% (*p* = 0.183), while in sequence B, they were 78.9% ± 23.8% vs. 84.4% ± 21.4% (*p* < 0.01). In both sequences, the lower range of the confidence interval for compliance with edONS was greater than the non-inferiority threshold (for sequence A Δ_Comp_^A^ was 4.5% [95% CI, −2.0% to 10.0%], and for sequence, B Δ_Comp_^B^ was 5.6% [95% CI, −3.0% to 14.0%]). The total discarded cost for each ONS was higher for heONS than edONS, being the difference statistically significant in sequence B. BMI increased slightly and not significantly in both sequences, and the percentage of patients with severe malnutrition was reduced. The frequency of gastrointestinal symptoms was low for both sequences, and satisfaction with ONS was slightly higher for edONS.

**Conclusion:**

Our findings highlight that edONS was non-inferior to heONS in terms of consumed energy over the prescribed, with a lower amount of edONS discarded, which suggests a higher efficiency of edONS.

## 1. Introduction

The primary cause of malnutrition in developed countries is disease (1). Disease-related malnutrition (DRM) is a prevalent condition, ranging between 20 and 50% in the hospital setting (2–4), and a major health public problem with high costs associated (4, 5). DRM can be triggered by a disease-specific inflammatory response as in cancer or major surgical procedures or linked to non-inflammatory etiologic mechanisms such as intestinal disorders (6, 7).

Increased daily nutritional needs (8), decreased intake and inadequate absorption of nutrients can result in a loss of weight and muscle mass. Malnutrition leads to a poor prognosis and treatment outcome (longer hospital stay, readmissions, infections, increased risk of chemotherapy-induced toxicity, postoperative complications and mortality), reduced functional status and health-related quality of life (2, 3, 9).

Clinical guidelines recommend performing nutritional assessment in all patients identified as at risk of malnutrition. For them, a personalized nutritional care plan should be established (6, 10). In order to meet the energy and protein requirements, this plan can include dietary advice, the treatment of symptoms impairing food intake, and offering oral nutritional supplements (ONS) (10). ONS have been shown to be effective in the treatment of DRM. However, compliance with intake is an important aspect to consider in order to achieve nutritional treatment goals and reduce the amount of product waste (11).

Although evidence has shown that, in general, adherence to ONS is adequate, there is a high proportion of patients in whom compliance is not optimal for meeting energy and nutritional requirements (11). Product-related factors such as energy density or prescribed volume should be taken into account in nutritional management as they may have an impact on compliance. Although a previous study suggests that consumption of energy-dense ONS (2.4 kcal/mL) results in a higher total energy and protein intake than the use of standard hypercaloric ONS (1.5–2.0 kcal/mL) (12), this study included a small sample of patients with a short follow-up of compliance, therefore more evidence is needed.

Currently, different hypercaloric ONS are available in Spain as nutritional support in DRM; however, only those with an energy density of no more than 2.1 kcal/ml are funded by the Spanish Health System (13). Evidence on the use of supplements with a higher energy density than currently funded is needed, so this pragmatic trial was carried out to compare compliance with two ONS, one with a high energy density (2.4 kcal/mL) and another hypercaloric one used as a reference (2.0 kcal/mL), in patients with DRM in different clinical situations. We hypothesized that compliance with high energy density ONS would be at least non-inferior to compliance with lower energy density ONS, with less product waste in the former.

## 2. Materials and methods

### 2.1. Study design and participants

This is a randomized, open-label crossover trial conducted in two Spanish tertiary hospitals in outpatients with DRM who required ONS. The protocol was approved by the Provincial Ethics Committee of Málaga (protocol code: NUT-ADHR-2.4; date of approval: 03/05/2019), and written informed consent was obtained from the patients. The protocol for this study was registered in ClinicalTrials.gov (NCT05609006).

The study included two 8-week sequences during which compliance with an energy-dense ONS (edONS; Fortimel Compact Protein®; Nutricia, Danone, Madrid, Spain; 2.4 kcal/mL) was compared with a high-energy ONS as a control/reference (heONS, Fortimel Extra®; Nutricia, Danone, Madrid, Spain; 2.0 kcal/mL), each for a period of 4 weeks in random order. The nutritional composition of both products is shown in Supplementary Table 1. Patients were randomly assigned to study sequences: edONS + heONS (sequence A) or heONS + edONS (sequence B). Since the nutritional status of the patient could be affected in case of temporary interruption, and because the carryover effect was not considered to have an impact on the measure of compliance, a washout period was not programmed between study periods.

Patients were eligible for inclusion in the trial if they were 18 years of age or older, presented with malnutrition or suspected malnutrition according to the Subjective Global Assessment (SGA categories B and C), had a high energy requirement and therefore needed the intake of two bottles/day of an ONS (≥2 kcal/mL) for a minimum period of 8 weeks. They were included if they were in any of the following situations: oncological patients who did not undergo surgery during the month prior to inclusion, including head and neck, esophagus, stomach, pancreas, or colon cancer; surgical patients who underwent surgery less than 1 month, including all types of surgical processes; and other non-surgical patients diagnosed with benign esophageal stricture, chronic radiation enteritis, and non-oncological maxillofacial lesions, cystic fibrosis, human immunodeficiency virus (HIV), malabsorption syndrome, ulcerative colitis, Crohn’s disease, fistula, intestinal pseudo-obstruction, chronic obstructive pulmonary disease (COPD), congestive heart failure (CHF), or who were scheduled to major surgery or transplantation within a period of no less than 2 months until inclusion. Except for surgical patients, and according to the site’s standard procedures, patients with other conditions should not have received a supplement during the month prior to inclusion. All included patients voluntarily agreed to participate in the study and give their signed consent for participation.

Patients were excluded if they suffered from chronic kidney disease or diabetes mellitus, required enteral tube feeding or parenteral nutrition, had any allergy or intolerance to the components of the study products, or had a scheduled surgery during the study period. Based on the physician’s opinion, patients were also excluded if they were unable to adhere to the protocol instructions, including lack of ability of the patient/caregiver to make use of the patient-directed study electronic case report form, and unable to complete the 8 weeks of study follow-up.

### 2.2. Procedures

Eligible patients were allocated in a 1:1 ratio to sequence A or B. Randomization was performed by a centralized computer-generated randomization service (sealed envelope™). To balance factors that could affect study outcomes, patients were stratified with a permuted block randomization method of blocks of size four according to their age (≤65 or >65 years) and their clinical condition (oncological, surgical or others).

After the allocation, patients were instructed to consume 2 bottles/day (morning and afternoon) of edONS (sequence A) or heONS (sequence B) for 4 weeks at home. In order to reduce taste fatigue, patients received ONS with two different flavors (strawberry and vanilla). In week 4, patients came to the hospital for nutritional assessment and were prescribed 2 bottles/day of the other product for a further 4 weeks. After 8 weeks, they came to the hospital for nutritional assessment. They could continue medical nutrition therapy as per standard practice if needed. Whenever possible the visits were face-to-face for the collection of the patient’s weight. However, due to pandemic restrictions, some patients were unable to come to the center and reported the weight obtained on home or community pharmacy scales.

### 2.3. Data collection

Sociodemographic (age and gender) and clinical data (main diagnosis, body mass index [BMI], nutritional status according to Subjective Global Assessment [SGA], and functional status according to Barthel Index for Activities of Daily Living) were collected by the investigators at the time of the inclusion (baseline visit) using an electronic case report form (eCRF). Two follow-up visits were established in weeks 4 (visit 1) and 8 (visit 2) to collect nutritional and functional status.

Patients daily collected the amount of product left over from the two intakes (morning and afternoon), through a patient’s electronic form sent to their smartphones (Supplementary Figure 1). To indicate the correct amount, patients were provided with a measuring cup to pour the leftover product to facilitate the completion of the form. In addition, to verify the amount indicated, patients were asked to photograph the measuring cup whenever possible and to record the picture together with the form.

Moreover, patients weekly collected information regarding gastrointestinal tolerance using the patient’s electronic form. To minimize a possible carryover effect between periods, gastrointestinal tolerance was registered by the patient on weeks 2, 3, and 4 of each period.

Lastly, patients’ satisfaction with the ONS was collected by the same method at the end of each period.

### 2.4. Outcomes

The primary outcome was the compliance rate (%) for each period defined as the percentage of consumed energy over the prescribed. From the daily amount left over and the amount consumed, the number of kcal consumed vs. prescribed kcals were estimated to obtain the compliance rate.

Secondary outcomes included changes in nutritional and functional status according SGA categories and Barthel Index, respectively. Other outcomes were gastrointestinal tolerance and satisfaction with ONS. Tolerance was measured using a numeric rate scale (NRS) of the frequency of symptoms (0, not at all; 10, very frequently) for the last 7 days such as nausea, vomiting, diarrhea, constipation, acid reflux, abdominal pain, bloated belly, stomach pain, flatulence, and satiety.

Satisfaction with the ONS taste, satiety, ease of completing the intake, and overall satisfaction were measured using an NRS of the level of satisfaction (0, very dissatisfied; 10, very satisfied). The mean score given by patients in both sequences to each ONS was estimated, regardless of the period.

### 2.5. Sample size predetermination

The primary analysis was designed to test whether compliance with edONS was non-inferior to heONS. Non-inferiority would be shown if the lower limit of the 95% confidence interval (CI) for the between-periods difference in the primary outcome was more than −5% (i.e., the difference between compliance with edONS and heONS for each sequence). This estimation is equivalent to one-sided noninferiority testing with an alpha of 0.05. Our original intention was to enroll 40 patients per sequence, which given a standard deviation of 15, would have provided 80% of power at an alpha level of 0.05, assuming 20% for possible dropouts.

However, enrollment proved much slower than expected due to the pandemic, and although the estimated sample was reached, drop-outs from the study were more frequent than expected. Thus, recruiting was stopped after at least 31 patients had been included into each sequence to be analyzed. This smaller sample reduced the power to test non-inferiority to 78%.

### 2.6. Statistical analysis

**Figure 1:**
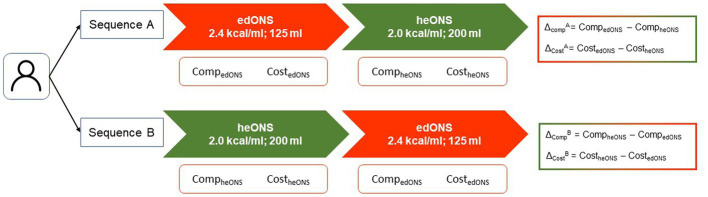
Study outcomes.

Using an intention-to-treat approach, we performed the primary analysis including all the patients who had undergone randomization and received ONS for at least 1 week in each period. For each sequence, the mean compliance with ONS was determined in both periods and the difference was calculated (Figure 1).

First, continuous variables were tested for normality using the Shapiro–Wilk test evidencing non-normality. As they are paired samples, compliance between periods was compared using Wilcoxon signed-rank test. Compliance between arms for first and second period was compared using Kruskal-Wallis test. Non-inferiority of edONS was calculated based on the mean difference of compliance and its CI for each sequence (Δ_Comp_^A^ and Δ_Comp_^B^), to test the lower limit of the 95% CI was greater than the non-inferiority limit established (−δ = −5%).

Additionally, a cost analysis was performed to estimate the cost of product discarded in each period per sequence by multiplying the average energy (kcal) of ONS not consumed by the cost per kcal (€, Spain; see Table 1). Wilcoxon signed-rank test was used to determine whether there were significant differences between periods in each arm.

**Table 1:** Cost of each product per bottle, volume, and kcal.

	edONS	heONS
Volume per bottle (mL)	125 mL	200 mL
Energy per bottle (kcal)	300 kcal	402 kcal
Cost per bottle	1.75 €	1.99 €
Cost per ml	0.014 €	0.010 €
Cost per Kcal	0.0058 €	0.0050 €

Unit costs as provided by Nutricia, Danone, Madrid, Spain.

All statistical analyses were performed using the software STATA v.14 (Stata Corp, College Station, TX, United States).

## 3. Results

### 3.1. Study participants and baseline characteristics

**Figure 2:**
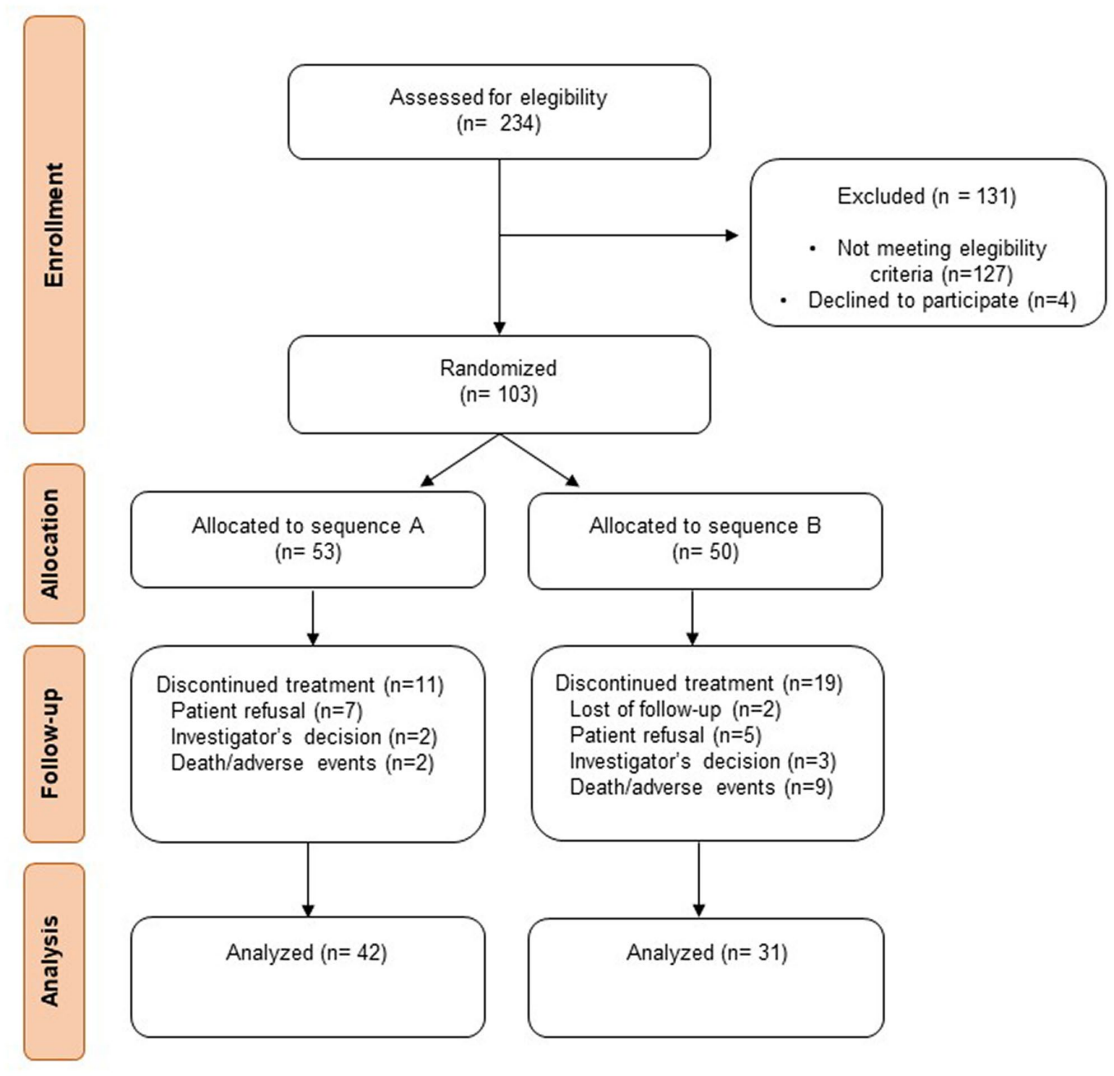
Flow diagram of the participants’ allocation.

From July 2019 to December 2021, a total 234 patients were screened across the two hospitals, and 103 were randomized (Figure 2) being 53 patients assigned to sequence A and 50 to sequence B.

The baseline characteristics of the patients are summarized in Table 2. The mean age was 55.7 ± 13.9 years, 37.0% were female, most of them being oncology patients (67.1%).

**Table 2:** Key demographic and clinical characteristics of the study population.

Patient characteristics	Sequence A (*N* = 42)	Sequence B (*N* = 31)	*p*
Age—year, mean ± SD	56.2 ± 13.1	55.1 ± 15.1	0.7208
**Sex,** ***n*** **(%)**			
Male	25 (59.5)	21 (67.7)	0.472
Female	17 (40.5)	10 (32.3)	
**Clinical condition,** ***n*** **(%)**			
Oncological patient	27 (64.3)	22 (71.0)	0.569
	6 (14.3)	2 (6.5)	
Surgical patient	9 (21.4)	7 (22.6)	
Other patients			
**Main diagnoses,** ***n*** **(%)**			
Head and neck cancer	9 (21.4)	6 (19.4)	--
Colorectal cancer	8 (19.0)	1 (3.2)	
Crohn Disease	5 (11.9)	2 (6.5)	
Stomach cancer	3 (7.1)	3 (9.7)	
Pancreatic cancer	3 (7.1)	1 (3.2)	
Lung cancer	1 (2.4)	3 (9.7)	
Breast cancer	2 (4.8)	1 (3.2)	
Skin cancer	1 (2.4)	2 (6.5)	
COPD	1 (2.4)	2 (6.5)	
Cervical cancer	1 (2.4)	1 (3.2)	
Malabsorption syndrome	1 (2.4)	1 (3.2)	
Cystic fibrosis	1 (2.4)	1 (3.2)	
Germ cancer	1 (2.4)	1 (3.2)	
HIV	1 (2.4)	1 (3.2)	
Liver cancer	2 (4.8)	-	
Brain tumor	-	2 (6.5)	
Esophageal cancer	1 (2.4)	-	
Chronic lymphocytic leukemia	1 (2.4)	-	
Pancreatic insufficiency	-	1 (3.2)	
Bile duct cancer	-	1 (3.2)	
Rectal cancer	-	1 (3.2)	
BMI, mean ± SD	22.1 (3.5)	21.9 (4.1)	0.444
**Classification according to BMI,** ***n*** **(%)**			
Low weight (BMI < 18,5)	8 (19.0)	7 (22.6)	--
Normal weight (18.5 < BMI < 25)	27 (64.3)	18 (58.1)	
Overweight (25 < BMI < 30)	6 (14.3)	4 (12.9)	
Obesity (BMI ≥ 30)	1 (2.4)	2 (6.5)	
**Nutritional status according to SGA,** *** n*** **(%)**			
Suspected malnutrition/moderate malnutrition (cat. B)	20 (47.6)	16 (51.6)	0.78
Severe malnutrition (Cat. C)	22 (52.4)	15 (48.4)	
**Functional status according Barthel index,** ***n*** **(%)**			
Independency (score 100)	35 (83.3)	26 (83.9)	0.809
Low dependency (score 91–99)	1 (2.4)	2 (6.5)	
Moderate dependency (score 61–90)	6 (14.3)	3 (9.7)	
Severe dependency (score 21–60)	-	-	
Total dependency (score ≤ 20)	-	-	

BMI, body mass index; COPD, chronic obstructive pulmonary disease; HIV, human immunodeficiency virus; SGA, subjective global assessment.

### 3.2. Compliance with ONS

**Figure 3:**
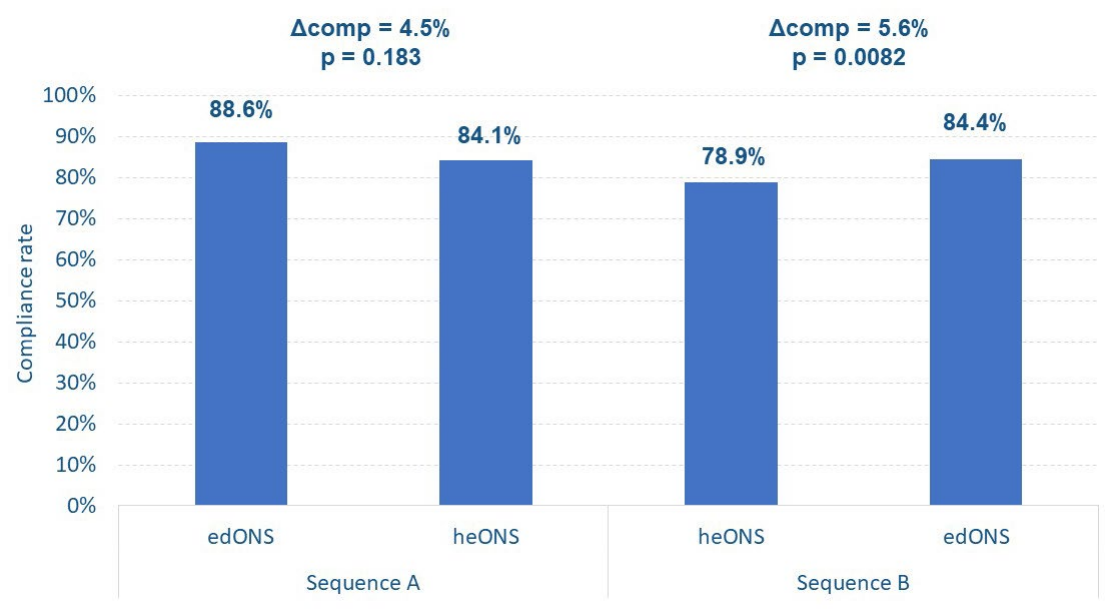
Compliance with the ONS in each study sequence.

Patients in sequence A recorded compliance with edONS and heONS a mean of 27.5 ± 1.6 and 25.1 ± 5.5 days, respectively. In this sequence, no significant differences in the compliance rate between periods were found (88.6% ± 14.3% vs. 84.1% ± 21.8%; *p* = 0.183; Figure 3).

Mean energy intake with edONS and heONS in sequence A was 532 and 634 kcal/day, respectively.

On the other hand, in sequence B, patients recorded compliance with heONS and edONS a mean of 27.3 ± 2.1 and 27.4 ± 2.5 days, respectively, showing significant differences in the compliance rate between periods (78.9% ± 23.8% vs. 84.4% ± 21.4%; *p* < 0.01; Figure 3). Mean energy intake with heONS and edONS in sequence B was 676 and 507 kcal/day, respectively.

Comparing the compliance with the first ONS received in each sequence, i.e., up to week 4 of treatment (period 1), the mean compliance with edONS was significantly higher than with heONS (88.6% ± 14.3% vs. 78.9% ± 23.8%; *p* = 0.0687).

**Figure 4:**
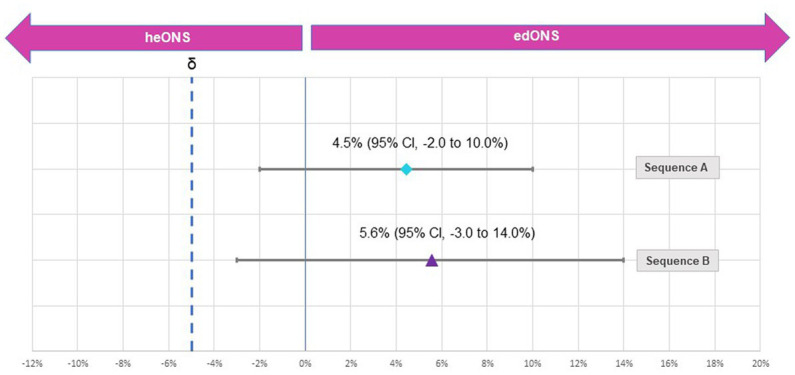
Difference in the compliance rate between periods for study sequences.

According with the non-inferiority analysis, in both sequences A and B, the lower range of the CI for compliance with the edONS was greater than the non-inferiority threshold, so it can be established that the edONS was non-inferior to the heONS (Figure 4). For sequence A, the Δ_Comp_^A^ was 4.5% (95% CI, −2.0% to 10.0%), and for sequence B, the Δ_Comp_^B^ was 5.6% (95% CI, −3.0% to 14.0%).

### 3.3. Nutritional and functional evolution

**Figure 5:**
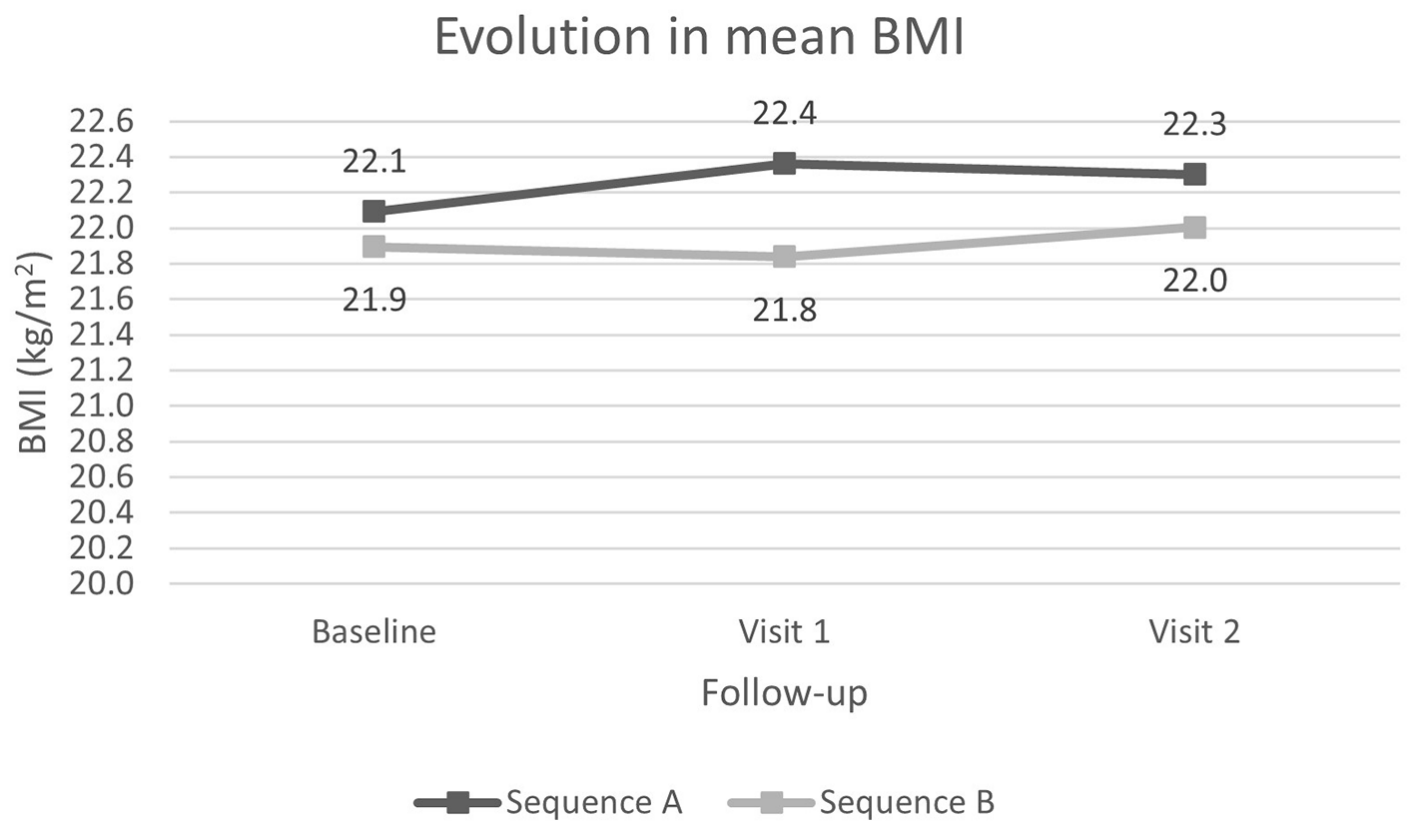
Evolution of the mean BMI from baseline visit over the course of the study.

BMI remained stable throughout follow-up within each sequence, increasing slightly from baseline to the final visit (Figure 5), although not significantly.

**Figure 6:**
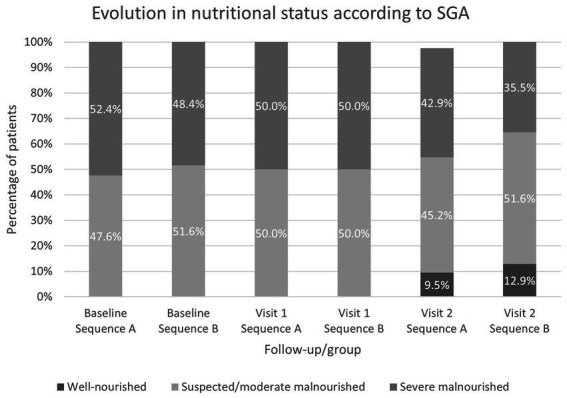
Evolution of the nutritional status according to SGA from baseline visit over the course of the study.

At 4 and 8 weeks, the percentage of patients with severe malnutrition according to SGA was reduced (Figure 6). However, there was little change in functional dependency status, with a slight increase in the moderate-highly dependent patients in sequence A at 8 weeks, from 6 patients (14.3%) at the baseline to 8 patients (19.2%) at visit 2.

### 3.4. Gastrointestinal tolerance and satisfaction

Overall, patients in both sequences perceived the frequency of occurrence of gastrointestinal symptoms to be low, being slightly higher in sequence B, mainly in symptoms such as abdominal pain and flatulence, but similar between the two periods within each sequence (Supplementary Table 2). Patients were more satiated with heONS in both sequences, with a greater numerical difference in the first period between sequences (Supplementary Figure 2). The mean scores on the satisfaction questions show a no statistically significant but slightly higher satisfaction obtained for the edONS in terms of taste, satiety, ease of finishing the supplement and overall satisfaction (Supplementary Figure 3).

### 3.5. Cost analysis

The mean ± SD total discarded cost for each sequence was: for sequence A, €10.78 ± 13.49 in the first period (edONS) and €15.20 ± 22.72 in the second period (heONS), with no statistically significant difference in discarded cost between periods of -€4.43 ± 19.20 (*p* = 0.1768); for sequence B, the discarded cost was €23.28 ± 26.48 in the first period (heONS) and €14.60 ± 20.56 in the second period (edONS), with a statistically significant difference in discarded cost between periods of -€8.68 ± 24.05 (*p* = 0.0431).

## 4. Discussion

This is the first randomized trial comparing the compliance with a low-volume energy-dense ONS of 2.4 kcal/mL and other standard-volume high-energy ONS of 2.0 kcal/mL conducted in malnourished or at risk of malnutrition patients in Spain.

Unlike in other countries, there is no funding by the Spanish Health System for ONS with a higher density of 2.1 kcal/mL, so the results of our study could be of particular interest both to the scientific community in general and to our health system in particular.

This study shows a high compliance with both ONS in the community setting, although slightly lower than that observed with energy-dense ONS as reported in previous studies (11).

Our findings highlighted that compliance with edONS was non-inferior to heONS, which confirms our research hypothesis. As this was a crossover trial, the patient was his or her own control, and although there were no statistically significant differences in compliance between ONS in sequence A, they were found in sequence B. These differences could be a consequence of intake fatigue. Even though intake fatigue may be associated with taste fatigue (14), our population received ONS with two different flavors to combine as preferred by the patient. Therefore, in our study it could be due to the prolonged consumption of the highest volume ONS during the first period of sequence B. It suggests that starting nutritional treatment with a low-volume ONS could reduce intake fatigue throughout the treatment period. Future studies should be carried out to test this hypothesis.

A previous study comparing low-volume high-energy ONS (2.4 kcal/mL) and a standard ONS (between 1.5–2.0 kcal/mL) in older people at risk of malnutrition showed a significantly higher compliance with the first one (12). Firstly, patients received the standard ONS in addition to their diet for 3 days, achieving an overall mean percentage of compliance of 77%, and then they received the low-volume high-energy ONS for 4 days, with a compliance of 91%. Although this study involved a short period of time, the results are in line with those found in the sequence B of our research (79% vs. 84%). Another study investigating the effects of energy-dense ONS vs. standard ONS of 1.5 kcal/mL in pediatric population, showed similar results with a greater proportion of patients with high compliance in the group receiving the energy-dense ONS (15). The authors attribute this to the good acceptability, and higher energy and nutrient density of the formula in a smaller volume.

Other studies have shown that volume and energy density could affect to nutritional intake, suggesting that small volume and energy-dense ONS may be an effective treatment for optimizing nutritional outcomes (11, 16). Our findings indicate that both ONS provide an acceptable daily caloric intake of more than 500 kcal/day. In fact, the nutritional outcomes in both sequences were similar, with a reduction in the proportion of patients with severe malnutrition at the end of the two follow-up periods. Although no significant differences in weight were found between baseline and final visit, it is important to note that many of the patients included in the study were oncology patients, and in this population, weight maintenance could be already a goal of nutritional treatment. Nevertheless, in the periods when patients took the more energy-dense ONS (2.4 kcal/mL), there was a tendency for BMI to increase, which was not the case with the ONS 2.0 kcal/mL.

As the amount of product discarded vs. prescribed was higher with heONS, this had a direct impact on cost, with the cost of discarded product being higher in this case, suggesting that edONS is more efficient, providing adequate caloric intake with a lower amount of product discarded because of higher compliance. This becomes even more important considering that many of the nutritional treatments are chronic, and therefore the funding and reimbursement of these ONS would represent a considerable saving for the national health system.

Additionally, both ONS showed adequate gastrointestinal tolerance in our study population. Within each sequence, the frequency of symptoms was similar between periods being low in all of them. It may indicate that in those patients with a higher frequency of symptoms, it may be associated with the main diagnosis. Moreover, satisfaction with both ONS was similar. Satiety with edONS was slightly lower than heONS, and the former was more ease to finish than the latter.

The study has several strengths. One of the main strengths is the multicentre and pragmatic character of the study, including patients from two centers belonging to different geographical areas in Spain, who required ONS as established in the usual clinical practice. On the other hand, the study was not restricted to patients with a single clinical situation (oncological, surgical and other non-surgical patients), which allows for the extension of the results to different pathologies.

Some study limitations need to be acknowledged. Firstly, the study was conducted throughout 2020, when the COVID-19 pandemic occurred, which influenced patient enrollment and resulted in not reaching the preliminary expected sample, but fortunately the results could be confirmed with significant statistical power. Secondly, both products not only differed on energy density but in volume too, which could make an influence on the comparison of them. On the one hand, due to the difference in volume of the two study ONS bottles, blinding to the interventions was not possible. On the other hand, each provided a different caloric intake, so it would be expected that their nutritional effects would be different, with a greater contribution from heONS. However, an intake of at least 500 kcal per day from each was considered adequate. Lastly, for the analysis of nutritional status, only BMI and nutritional assessment by SGA were taken into account. BMI may not reflect early changes in body composition with sufficient sensitivity, whereas results of SGA could be difficult to interpret in case of normal weight and obese patients (17). In addition, due to pandemic restrictions, at some visits several patients were weighed on different weighing scales which could lead to variations, albeit minimal, inpatient weight unrelated to nutritional intake. Future studies should include other nutritional measures or parameters to detect both changes in body composition and functionality.

## 5. Conclusion

Our findings highlight that the edONS was non-inferior to the heONS in terms of compliance defined as consumed energy over the prescribed, but with a lower amount of edONS discarded, which suggests a higher efficiency with the use of energy-dense ONS. EdONS may be a good alternative to other higher volume hyperprotein and hypercaloric formulas, which can help improve patient compliance while maintaining the nutritional status of patients malnourished or at risk of malnutrition.

## Data availability statement

The data presented in this study are available on reasonable request from the corresponding authors.

## Ethics statement

The studies involving human participants were reviewed and approved by Provincial Ethics Committe of Málaga. The patients/participants provided their written informed consent to participate in this study.

## Author contributions

ML-S and GO supervised and coordinated the study. ML-S, GO, and LL conceptualized the study and developed and coordinated the methodology. FL, MT, MG, MM-J, and MR performed the investigation. LL validated the data and conducted the formal analysis. All authors contributed to the article and approved the submitted version.

## Funding

The authors declare that this study received funding from Danone Specialized Nutrition. The funder was not involved in the study design, collection, analysis, interpretation of data, the writing of this article, or the decision to submit it for publication.

## Conflict of interest

LL works for an independent research entity, Outcomes’10, that received funding from Danone Specialized Nutrition to conduct the project and for medical writing.

The remaining authors declare that the research was conducted in the absence of any commercial or financial relationships that could be construed as a potential conflict of interest

## Publisher’s note

All claims expressed in this article are solely those of the authors and do not necessarily represent those of their affiliated organizations, or those of the publisher, the editors and the reviewers. Any product that may be evaluated in this article, or claim that may be made by its manufacturer, is not guaranteed or endorsed by the publisher.
