# Supplementary material for: Compliance with a high-protein and energy-dense oral nutritional supplement in patients with disease-related malnutrition: a randomized open-label crossover trial
Source: Front Nutr. 2023 May 2;10:1182445. doi: 10.3389/fnut.2023.1182445 (PMC10186345; doi:10.3389/fnut.2023.1182445)
Supplement: Supplementary file 1 [file Data_Sheet_1.docx]

Supplementary Material

**Compliance with a high-protein and energy-dense oral nutritional supplement in patients with disease-related malnutrition: a randomized open-label crossover trial**

**Miguel Leon-Sanz, Francisca Linares, Montserrat Gonzalo, María José Tapia, María Maiz-Jimenez, Marta Ruiz Aguado, Luis Lizán, Gabriel Olveira**

***Correspondence:**

Gabriel Olveira

[gabrielm.olveira.sspa@juntadeandalucia.es](mailto:gabrielm.olveira.sspa@juntadeandalucia.es)

Miguel Leon-Sanz

[mleon@h12o.es](mailto:mleon@h12o.es)

**Supplementary Table 1.** Macronutrient composition of the study products.

|  | edONS (125ml) | heONS (200ml) |
| --- | --- | --- |
| Energy density, kcal/ml | 2.4 | 2.01 |
| Energy, kcal | 300 | 402 |
| Protein, g (%) | 18 (24.0%) | 20.2 (20.1%) |
| Fat, g (%) | 11.8 (35.3%) | 17.2 (38.2%) |
| Carbohydrate, g (%) | 30.5 (40.7%) | 42.2 (41.7%) |
| Fibre, g | - | - |

**Supplementary Figure 1.** Image of the electronic patient form for the collection of product consumption.


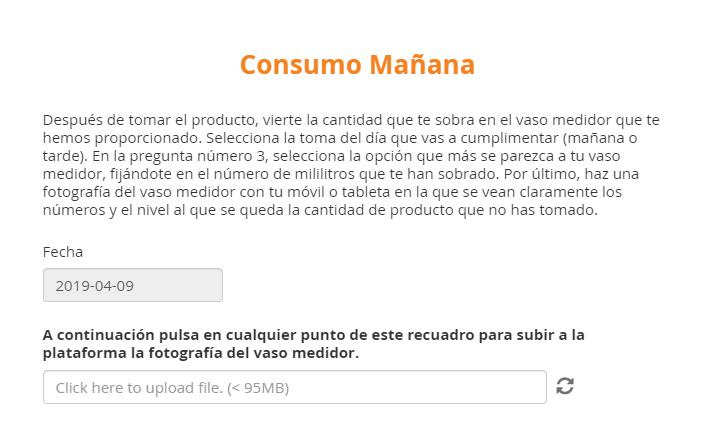


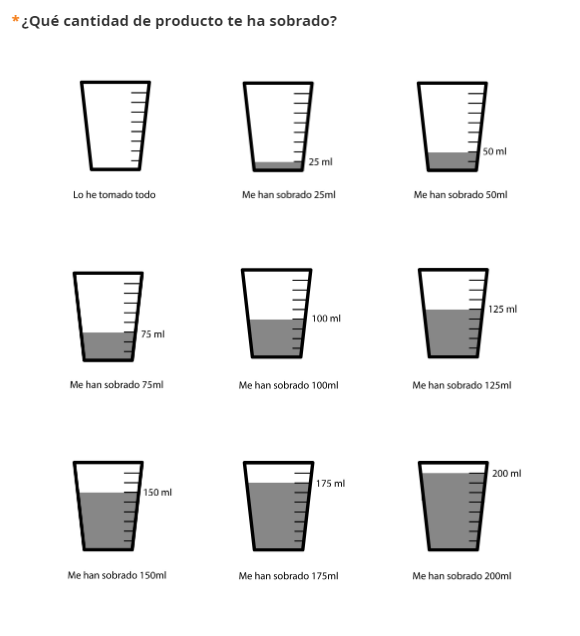


**Supplementary Table 2.** Patient’s perception of symptoms frequency during last 7 days (Not at all=0; very frequently=10).

| **In the last 7 days,** | **Sequence** | **Week 2** | **Week 3** | **Week 4** | **Week 6** | **Week 7** | **Week 8** |
| --- | --- | --- | --- | --- | --- | --- | --- |
| **did you experience nausea?** | **Sequence A** | 1.2 (2.3) | 1.6 (2.8) | 1.1 (2.2) | 1.9 (2.7) | 1.1 (2.4) | 1.6 (2.7) |
|  | **Sequence B** | 2.0 (2.7) | 2.0 (2.8) | 1.5 (2.7) | 1.5 (2.8) | 0.9 (1.7) | 1.1 (2.5) |
| **did you experience vomiting?** | **Sequence A** | 0.3 (0.8) | 1.1 (2.5) | 0.5 (1.5) | 1.1 (2.7) | 0.5 (1.6) | 1.0 (2.2) |
|  | **Sequence B** | 0.8 (2.2) | 0.4 (0.7) | 0.3 (1.0) | 0.3 (1.1) | 0.1 (0.3) | 0.7 (2.2) |
| **did you experience diarrhea?** | **Sequence A** | 0.6 (1.7) | 1.3 (2.5) | 0.9 (1.9) | 1.7 (2.9) | 1.3 (2.5) | 1.2 (2.7) |
|  | **Sequence B** | 1.4 (2.6) | 1.2 (2.6) | 0.8 (2.1) | 1.1 (2.1) | 1.0 (2.1) | 1.2 (2.8) |
| **did you experience constipation?** | **Sequence A** | 3.0 (3.3) | 3.4 (3.7) | 3.0 (3.5) | 2.7 (3.7) | 2.0 (3.1) | 2.0 (3.1) |
|  | **Sequence B** | 3.1 (3.6) | 2.5 (3.0) | 3.1 (3.5) | 2.6 (3.8) | 3.3 (3.5) | 2.8 (3.1) |
| **did you experience acid reflux?** | **Sequence A** | 1.0 (2.1) | 1.8 (3.0) | 1.5 (2.8) | 1.8 (2.8) | 1.6 (2.6) | 1.1 (2.5) |
|  | **Sequence B** | 1.8 (3.0) | 2.3 (3.4) | 1.7 (2.8) | 1.3 (2.4) | 1.5 (2.3) | 1.1 (2.4) |
| **did you experience abdominal pain?** | **Sequence A** | 0.8 (1.9) | 1.9 (2.8) | 1.2 (2.1) | 1.6 (2.8) | 1.5 (2.8) | 1.3 (2.6) |
|  | **Sequence B** | 2.7 (3.1) | 2.5 (3.3) | 2.1 (3.3) | 2.3 (3.1) | 2.2 (3.1) | 2.0 (2.9) |
| **did you experience bloated belly?** | **Sequence A** | 1.1 (2.2) | 1.5 (2.5) | 1.3 (2.3) | 1.9 (2.6) | 1.8 (2.9) | 1.4 (2.9) |
|  | **Sequence B** | 2.8 (3.5) | 2.2 (3.0) | 2.2 (2.7) | 2.2 (2.9) | 2.2 (3.1) | 1.9 (2.4) |
| **did you experience stomach pain?** | **Sequence A** | 0.8 (2.1) | 1.7 (2.9) | 1.3 (2.5) | 1.9 (3.0) | 1.3 (2.3) | 1.5 (2.8) |
|  | **Sequence B** | 1.3 (2.7) | 1.6 (2.8) | 1.3 (2.7) | 1.9 (2.8) | 1.5 (2.8) | 1.1 (2.2) |
| **did you experience flatulence?** | **Sequence A** | 2.1 (3.0) | 3.0 (3.3) | 2.9 (3.2) | 3.1 (3.2) | 2.5 (3.1) | 1.9 (2.7) |
|  | **Sequence B** | 4.0 (3.2) | 4.1 (3.7) | 4.1 (3.6) | 3.8 (3.2) | 3.4 (3.4) | 3.5 (3.0) |
| **did you feel satiated after taking the supplement?** | **Sequence A** | 5.2 (3.9) | 4.9 (3.9) | 5.1 (3.5) | 5.9 (3.6) | 5.8 (3.9) | 6.7 (3.5) |
|  | **Sequence B** | 7.0 (3.3) | 6.6 (3.5) | 7.2 (2.9) | 6.5 (3.3) | 6.6 (3.6) | 7.0 (2.9) |

**Supplementary Figure 2.** Patient’s perception of satiety frequency during last 7 days (Not at all=0; very frequently=10).

**
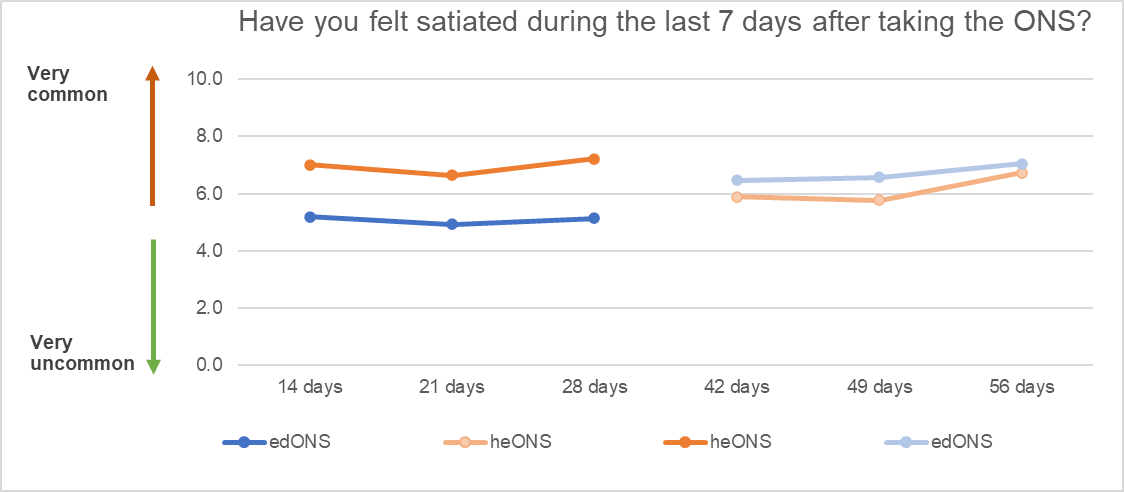
**

**Supplementary Figure 3.** Mean score for satisfaction questions according to the ONS consumed.

**
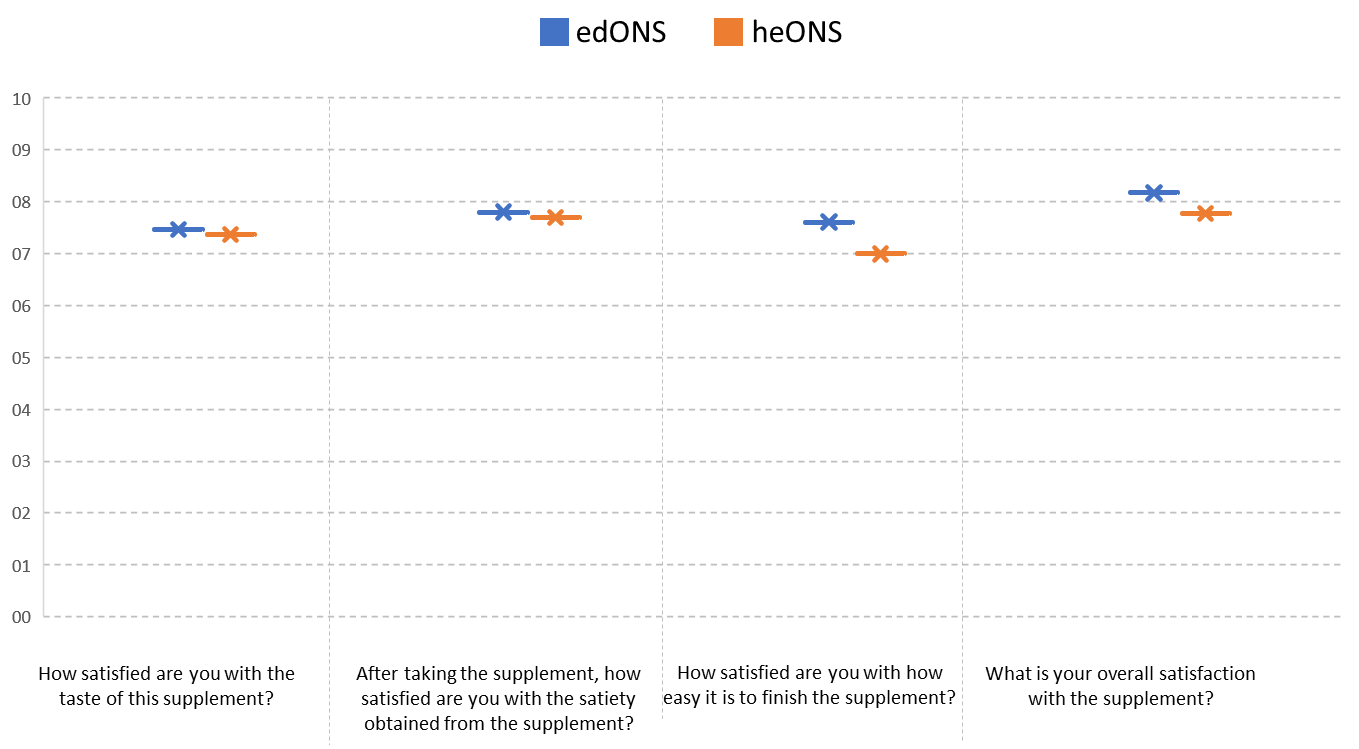
**
